# An Alternative Proposal for the Reaction Mechanism of Light-Dependent Protochlorophyllide Oxidoreductase

**DOI:** 10.1021/acscatal.1c05351

**Published:** 2022-02-07

**Authors:** Pedro J. Silva, Qi Cheng

**Affiliations:** † FP-I3ID/Fac. de Ciências da Saúde, Universidade Fernando Pessoa, 4200-150 Porto, Portugal; ‡ UCIBIO@REQUIMTE, BioSIM, Departamento de Biomedicina, Faculdade de Medicina, Universidade do Porto, 4200-319 Porto, Portugal; § Department of Biochemistry, College of Life Sciences, Hebei Agricultural University, Baoding, Hebei 071000, China; ∥ State Key Laboratory of North China Crop Improvement and Regulation, Hebei Agricultural University, Baoding, Hebei 071000, China

**Keywords:** time-dependent density functional theory, photoenzyme, photocatalysis, electron transfer, protochlorophyllide

## Abstract

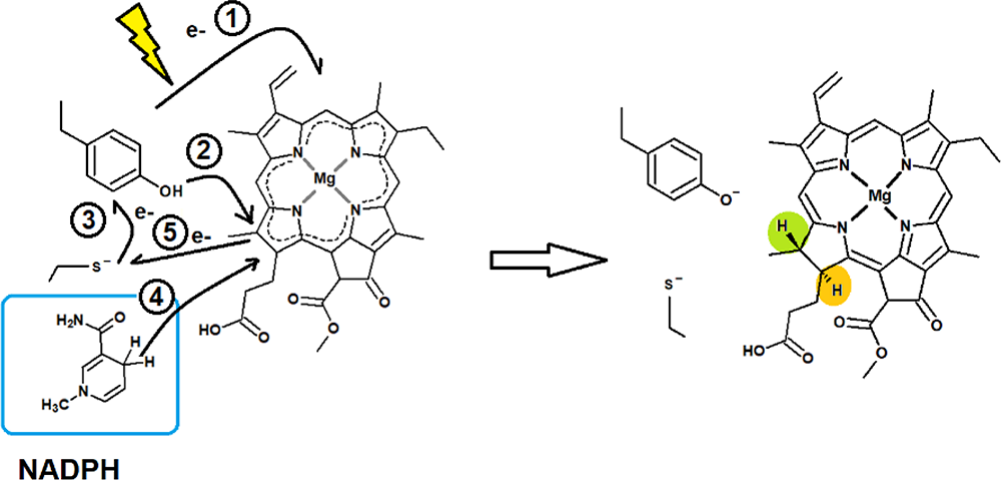

Light-dependent protochlorophyllide oxidoreductase is one of the few known enzymes that require a quantum of light to start their catalytic cycle. Upon excitation, it uses NADPH to reduce the C_17_–C_18_ in its substrate (protochlorophyllide) through a complex mechanism that has heretofore eluded precise determination. Isotopic labeling experiments have shown that the hydride-transfer step is very fast, with a small barrier close to 9 kcal mol^–1^, and is followed by a proton-transfer step, which has been postulated to be the protonation of the product by the strictly conserved Tyr189 residue. Since the structure of the enzyme–substrate complex has not yet been experimentally determined, we first used modeling techniques to discover the actual substrate binding mode. Two possible binding modes were found, both yielding stable binding (as ascertained through molecular dynamics simulations) but only one of which placed the critical C17=C18 bond consistently close to the NADPH *pro*-*S* hydrogen and to Tyr189. This binding pose was then used as a starting point for the testing of previous mechanistic proposals using time-dependent density functional theory. The quantum-chemical computations clearly showed that such mechanisms have prohibitively high activation energies. Instead, these computations showed the feasibility of an alternative mechanism initiated by excited-state electron transfer from the key Tyr189 to the substrate. This mechanism appears to agree with the extant experimental data and reinterprets the final protonation step as a proton transfer to the active site itself rather than to the product, aiming at regenerating it for another round of catalysis.

## Introduction

In all photosynthetic organisms, chlorophylls function as light-absorbing photopigments harvesting a range of light energy with various wavelengths. Chlorophyll biosynthesis recurs in similar ways in anoxygenic phototrophic proteobacteria as well as oxygenic phototrophic cyanobacteria, algae, and higher plants.^1−4^ In this pathway (chlorophyll biosynthesis), the two-electron reduction of the C_17_–C_18_ double bond in protochlorophyllide to chlorophyllide is a key step. Two distinctive versions of protochlorophyllide oxidoreductase (POR) enzymes are present in most photosynthetic organisms: a light-dependent version known as LPOR (absent, however, from anoxygenic photosynthetic bacteria) and a light-independent version known as “dark-operative POR” or DPOR (which is absent from angiosperms). These two enzymes are structurally and genetically different, and their evolutionary stories are fascinating.^2,5−10^ Phylogenetic analysis shows that LPOR first arose in cyanobacteria probably under strong evolutionary pressures to find an oxygen-insensitive enzyme capable of causing PChlide reduction.^2,9^ The gene was inherited by land plants from cyanobacteria ancestors, and now, LPOR is the only PChlide reductase in angiosperms. LPOR belongs to the short-chain dehydrogenase/reductase superfamily, whereas DPOR is, surprisingly, genetically and evolutionarily related to nitrogenase, a multi-subunit metalloenzyme capable of converting atmospheric nitrogen into ammonia. Despite relatively low identity at the amino acid level, the crystal structures of both nitrogenase^11−14^ and DPOR^15,16^ not only share overall structure similarities but also interact with related Fe–S proteins, which act as their electron donors upon ATP hydrolysis.^17,18^ Although extensive research on LPOR and DPOR has been carried out at the molecular level over the past two decades,^2,19−35^ the precise details of their mechanisms still remain insufficiently described. It is generally assumed that LPOR uses light absorbed by its substrate PChlide to prime its cofactor NADPH for hydride transfer from the *pro-S* face of its nicotinamide ring to the C_17_ position of the PChlide molecule, followed by a proton transfer reaction from a conserved Tyr residue to the C_18_ position of PChlide. Catalysis by LPOR is also thought to involve thermally excited protein dynamics that allow molecular motions to occur on an ultrafast timescale,^36^ which makes it unique from other members of the short-chain dehydrogenase superfamily. Structural studies have until recently been restricted to the insights obtained from homology modeling,^37−39^ but the publication of the first X-ray crystal structures of LPOR from both model cyanobacteria *Synechocystis* sp. PCC 6803 and *Thermosynechococcus elongatus* complexed with cofactor NADPH^40^ now allows a more well-grounded investigation of the precise molecular determinants of ligand binding and catalysis. However, unlike the DPOR–PChlide complex, the precise PChlide-binding site in the LPOR–NADPH complex is yet to be determined. In this manuscript, we describe the computational discovery of a promising binding mode, which we used as starting point for a quantum-mechanical exploration of the reaction mechanism. We expect the insights obtained from this research to offer fruitful venues for further mechanistic investigations and eventual adaptation of the LPOR framework to non-natural catalysis.

## Computational Methods

Docking and molecular dynamics were performed in YASARA.^41^ Substrate parameterization was performed with the AM1BCC protocol.^42,43^ All molecular dynamics simulations were run with the AMBER03 force field^44^ using a multiple time step of 2.5 fs for intramolecular and 5 fs for intermolecular forces. Simulations were performed in cells 15 Å larger than the solute along each axis (final cell dimensions: 83 Å × 79 Å × 77 Å), and counterions (45 Cl^–^ and 43 Na^+^) were added to a final concentration of 0.9% NaCl. In total, the simulations contained approximately 51,630 atoms. A 8 Å cutoff was taken for Lennard–Jones forces and the direct space portion of the electrostatic forces, which were calculated using the particle mesh Ewald method^45^ with a grid spacing of <1 Å, fourth-order B-splines, and a tolerance of 10^–4^ for the direct space sum. Simulated annealing minimizations started at 298 K, and velocities were scaled down by 0.9 for every 10 steps for a total time of 5 ps. After annealing, simulations were run at 298 K. Temperature was adjusted using a Berendsen thermostat^46^ based on the time-averaged temperature, i.e., to minimize the impact of temperature control, velocities were rescaled only about every 100 simulation steps whenever the average of the last 100 measured temperatures converged.

Since the available starting structure^40^ of the light-dependent protochlorophyllide reductase from *Synechocystis* (PDB: 6R48 , chain B) is in a closed conformation, PChlide was initially placed just outside the cleft lying between Ile153 and the 225–250 domain, oriented so that its C17 atom faced the NADPH *pro-S* hydrogen. The initial distance between these two atoms (which we joined with a spring with force constant 3 kcal mol^–1^ Å^–2^) was 14.5 Å. PChlide was then brought closer to the *pro-S* hydrogen through an umbrella sampling simulation by progressively decreasing the equilibrium distance of the spring by 1 Å every 3 ns. The structure we obtained after the simulation was more open than the starting structure (partly through conformational unraveling of the 225–250 domain), but it still did not afford a good docking pose. We used this structure as target for docking after replacing the unwound 225–250 domain with its initial (properly folded) structure while keeping it in the newly generated open orientation relative to the rest of the protein. Substrate docking was then performed with AutoDock Vina^47^ using default docking parameters. The docking region was confined to a 29.0 Å × 29.0 Å × 29.0 Å box centered on the crevice revealed by the umbrella sampling simulation. The side chains of the amino acids lining the crevice (Leu149, Lys152, Ile155, Tyr189, Lys190, Lys193, Tyr219, Cys222, Thr226, Leu228, Phe229, Tyr233, Phe236, Thr248, and Lys249) were kept flexible. One hundred docking runs were performed. The docking poses showing appropriate orientation and distances of the C_17_ and NADPH *pro-S* hydrogen atoms were then subjected to molecular dynamics simulations for at least 100 ns. In the initial 25 ns of each simulation, the NADPH-C17 and Tyr219–C_18_ distances were progressively shortened to prevent the escape of the ligand from the active site cleft. All restraints were removed at the time point of 25.000 ns.

The stable structure obtained after 300 ns of MD simulation was used as the starting point for the computation of the potential of mean force for ligand binding. The potential of mean force was obtained through umbrella sampling simulations performed by constraining the distance between the ligand Mg^2+^ atom and the Val14 amide nitrogen atom (which is located in the base of the identified pocket) with a harmonic potential of the form *V* = 1/2*k*(*x* – *x*_0_)^2^ with *k* equal to 14.0 kcal mol^–1 Å–2^. Sampling was performed in bins 0.7 Å apart for 6 ns per bin. In each bin, the first full ns was discarded from the analysis. The unbiased distributions were obtained through the weighted histogram analysis method (WHAM)^48,49^ using a bin size of 0.2 Å.

Density functional and time-dependent density functional computations were performed using the PBE0 functional,^50,51^ which has been shown to provide good results when applied to the analysis of singlet, triplet, valence, and Rydberg excited states in combination with large basis sets, returning a mean absolute error of 0.28 eV (the lowest among global hybrids tested).^52^ The size of our system prevents the use of such large basis sets, but preliminary computations with basis sets of different sizes showed that in our PChlide+NADPH+Tyr189 system, the 6-31G(d) basis set yielded the same excitations as those obtained with a larger number of polarization functions and/or diffuse functions and afforded excitation energies within 0.1 eV of the energies computed with those larger basis sets. All TDDFT computations and geometry optimizations were therefore performed with the 6-31G(d) basis set. Between 8 and 10 excited states were computed in each TDDFT run. Although TDDFT is known to underestimate the energies of excitations with charge-transfer character, this limitation is not expected to qualitatively affect our results, since the relevant charge-transfer state we detected (see below) geometrically relaxes into a deprotonated state without any energetic barrier; any effect of the underestimation of the original excited state energy is therefore expected to, at most, provide an even more spontaneous relaxation into that state.

Due to the extremely high computational cost of DFT and TDDFT computations in large systems, we performed our computations in suitably truncated models with varying sizes. All models included the full protochlorophyllide (with the propionate group replaced by an ethyl substituent) and either the nicotinamide ring of NADPH or the complete side chain of Tyr189. The model systems were saturated with hydrogen atoms, which replaced the NH- and C=O groups removed from Tyr189 and the *O*-ribosyl removed from NADPH. Several water molecules (either bridging the Tyr189 with C_18_ or solvating the amide moiety of the nicotinamide) were also included. Larger models included both the nicotinamide ring and Tyr189. To prevent the truncation of the system from allowing unrealistic movements of the NADPH/Tyr189/PChlide moieties, several atoms were kept frozen during the optimizations: the central Mg, three atoms in the PChlide ring, the Cα and Cβ atoms of Tyr189, and the nicotinamide methyl carbon atom, which connects to the rest of the NADPH molecule in the protein structure. Coordinates taken from autogenerated delocalized coordinates^53^ were used for geometry optimizations. Transition-state structures from ground-state potential energy surfaces were confirmed by checking that a single imaginary frequency was present, corresponding to a vibration connecting the reactant and product states. ZPVE and thermal effects were computed at 298.15 K using scaling factor of 0.9726^54^ for the computed vibrational frequencies. Single-point energies of the DFT-optimized geometries were then calculated using the same functional using the 6-311G(d,p) basis set supplemented with diffuse functions on non-hydrogen atoms. Due to extensive linear dependence, SCF convergence could only be reached after removal of the diffuse functions from the carbon atoms that were part of the porphyrin-delocalized π-system. This basis set will be referred to herein as 6-311G(partial+)(d,p). All ground-state energy values described in the text include solvation effects (ε = 10) computed using the polarizable continuum model^55−57^ implemented in Firefly. Quantum chemistry computations were performed with the Firefly^58^ quantum chemistry package, which is partially based on the GAMESS (US)^59^ source code.

For reactions in excited states, computational cost prevented us from computing exact transition states; in these cases, we built the corresponding potential energy surfaces (PES) by sequentially constraining the bond length of interest (in 0.10 Å increments) and performing full optimization subject to those constraints. Each PES contained a single maximum between the reactant and product states, which was selected as an appropriate approximation of the corresponding transition state. The excited state-tracking feature present in Firefly was used to ensure that the correct excited state was kept when constraining the reaction coordinate to successively larger/smaller values. Activation energies of electron transfer between amino acid side chains and Tyr189/PChlide radicals were computed using a Marcus theory formalism:^60^ briefly, the geometries of all species thought to be involved in one-electron transfer were separately optimized in both oxidation states, the inner-shell electron-transfer reorganization energies were then computed by using the oxidized geometry for the reduced state (and vice versa), and finally, Marcus parabolas were built from these values to ascertain the energy at their intersection. Electron transfer rates were computed from these values as described by Moser and Dutton^61^ using an adaptation of the equation , where *R* is the intercenter distance (in Å), Δ*G* is the change in energy between reactants and products (in eV), and λ is the reorganization energy (in eV). The factor  corresponds to the activation energy of an electron transfer where the reorganization energy of the product state is equal to the reorganization energy of the reactant state, and when it is replaced by the more accurate activation energy computed using the Marcus parabolas built using the different reorganization energies of each state (as described above), that expression becomes log*k*_ET_ = 15.0 – 0.6*R* – 12.4Δ*G*_act_. For some systems (deprotonated Tyr189 radical/deprotonated Cys222, C_18_-protonated one-electron-reduced PChlide/radical deprotonated Cys222, and one-electron-reduced Chlide/deprotonated Cys222 radical), the outer sphere (solvent) contribution to the reorganization energy was evaluated through the method described by Zhou and Szabo^62^ using the final 80 ns of 100 ns long MD simulations initiated at the 100 ns conformation of the PChOR:NADPH:PChlide ternary complex (after appropriate changes in protonation state/electronic charge). Briefly for each snapshot, the gap between the energy of the original electronic state and the energy of that same conformation with the product electronic distribution was computed. The average energy gap and variance of the energy gap, along with the intrinsic reaction energy in solution computed earlier by QM methods, were then used according to eqs 3.12, 3.15, and 4.20 in ref (62) to obtain the outer sphere contribution to the reorganization energy.

To ascertain the relative abundance of the different protonation or redox states of the amino acid side chains of the enzyme, continuum electrostatic calculations were performed using MEAD^63^ AMBER14 charges, and radii^64^ were assigned to the protein structure using YASARA. The solvent probe radius was 1.4 Å, which should provide a reasonable spherical approximation of the water molecule. The ionic exclusion layer thickness was set at 2.0 Å, and temperature was set to 300 K. The dielectric constant used for the solvent region was 80, the approximate value for bulk water at room temperatures. The dielectric constant for the protein interior was set to 15, the value previously found to yield optimum results with this method.^65,66^ A two-step focusing method was used. A first calculation using a 300 Å^3^ cube with a 1.0 Å lattice spacing centered on the protein was followed by a second calculation using a 75 Å^3^ cube with a 0.25 Å spacing centered on the titrable site. All Asp, Glu, His, Cys, Tyr, and Lys residues were allowed to titrate. The sampling of proton-binding states was done using the MCRP program (Monte Carlo for Reduction and Protonation), which implements a Monte Carlo method described by Baptista et al.^67^ Sampling was performed at 0.5 pH unit intervals in the 6–8 range using 10^7^ Monte Carlo steps. For redox titrations, deprotonated Tyr189 and deprotonated Cys222 were allowed to transition between their anionic and radical neutral forms. Sampling was performed from pH 6 to 8 at 0.4 pH unit intervals as well as in a redox potential window 800 mV wide at 20 mV intervals using 10^7^ Monte Carlo steps.

## Results

### Identification of the Substrate Binding Mode

**Figure 1:**
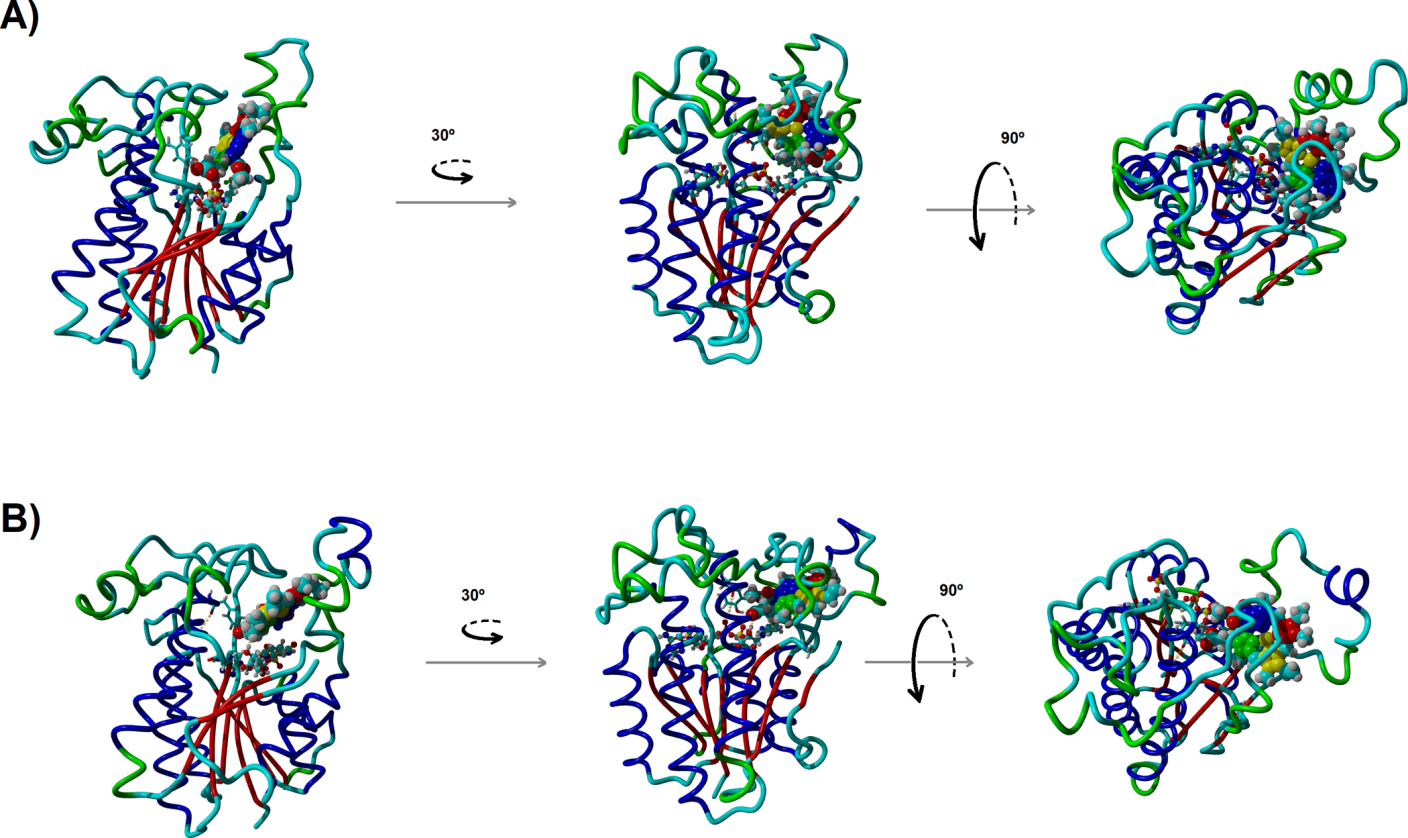
Comparison of the two binding modes of PChlide to LPOR. Both snapshots were taken 100 ns into each molecular dynamics simulation. (A) Tyr189 close to the reactive ring D. The propionic acid side chain on ring D interacts with the backbone of Val142. (B) Tyr189 interacts with the propionic acid side chain on ring D. Rings are color-coded for ease of reference: yellow, ring A; red, ring B; blue, rings C and E; and green, ring D.

**Figure 2:**
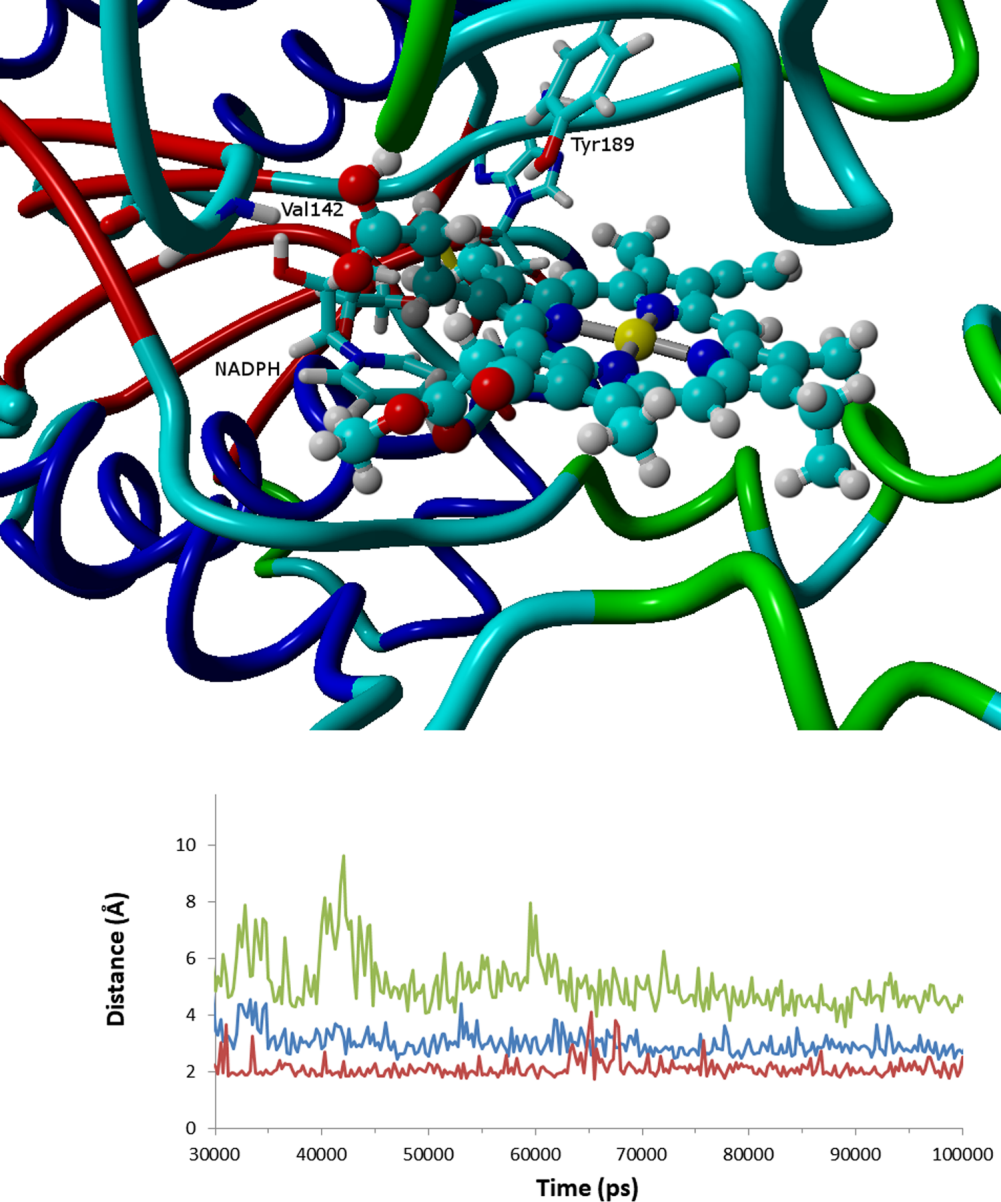
First stable binding pose. The graph depicts the time evolution of NADPH-C17 (blue), Val142 NH-propionate (red), and Tyr189–C_18_ (green) distances.

Molecular docking on the artificially opened cavity obtained through steered molecular dynamics revealed two plausible binding modes with appropriate NADPH-C17 distances. In both cases, however, the reactive C=C bond remained relatively far from the catalytically important Tyr189 and much closer to another tyrosine residue, Tyr219. Molecular dynamics simulations started from each of these positions converged into different conformations (Figure 1). The first pose (Figure 2) afforded a binding mode where one face of the protochlorophylide contacts the protein surface and the circumference of the substrate is almost completely accessible to the solvent. Portions of the flexible 225–250 domain partially shield the other face of the protochlorophyllide from the solvent: ring B is sandwiched between Pro154 and Leu228, and the ethyl substituent in its C8 atom lies in a pocket defined by Tyr233, Phe236, and Val247. The propionate group attached to protochlorophyllide C_17_ anchors the substrate in place through a strong interaction (2.11 ± 0.32 Å) with the amide nitrogen of Val142, and a short (3.00 ± 0.39 Å) C17-NADPH distance is maintained throughout. Tyr189 is properly placed in relative proximity (5.08 ± 0.92 Å) to the protochlorophyllide C_18_ atom separated from the C_18_ atom by a single water molecule, which can act as a proton relay. Computation of the potential of mean force for this binding mode yielded a value of 26.1 ± 0.7 kcal mol^–1^, which is far more favorable than the 8.4 kcal mol^–1^ computed for the binding mode computed by Zhang et al.^40^

**Figure 3:**
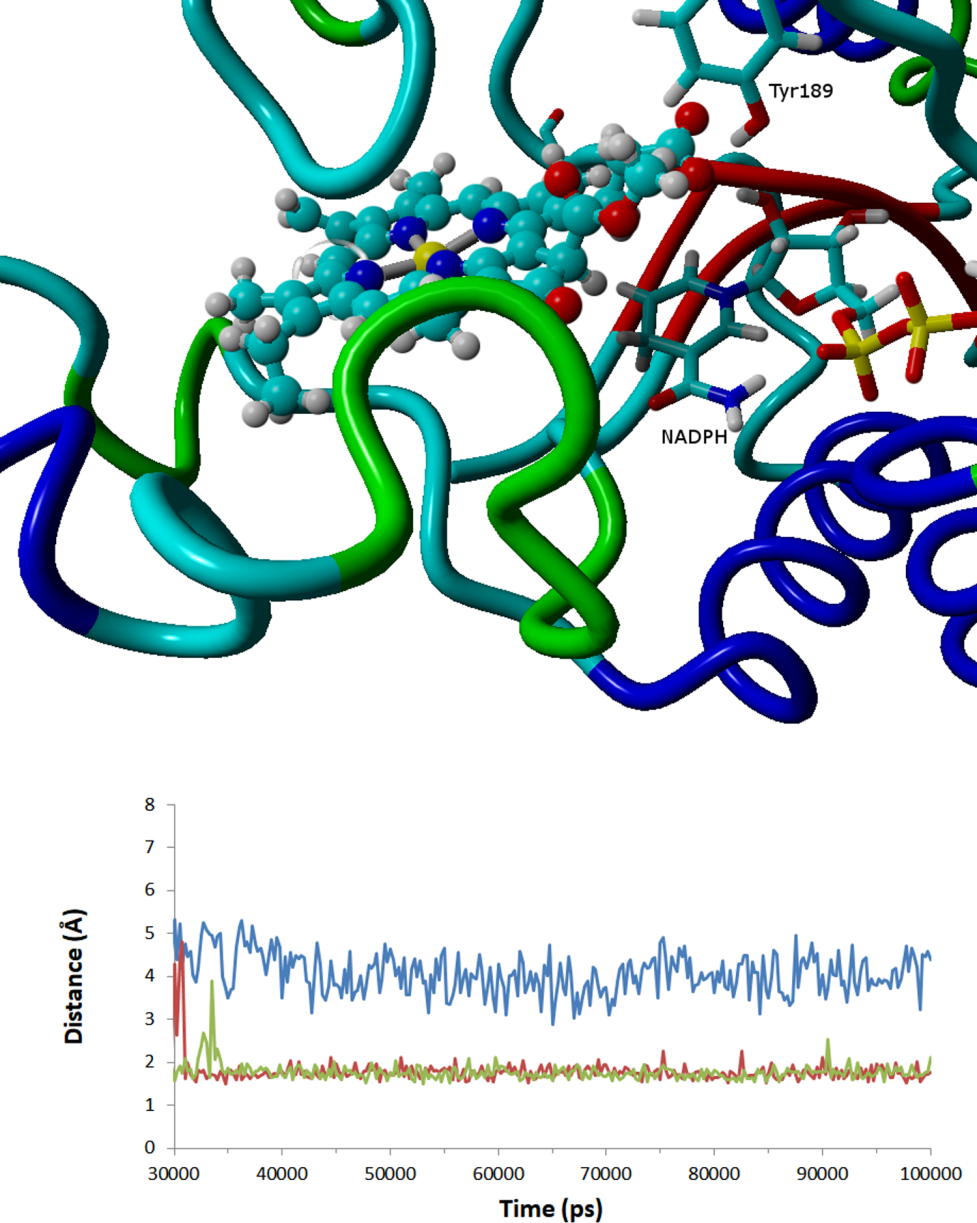
Second stable binding pose. The graph depicts the time evolution of NADPH–C_17_ (blue), propionate–ribose (green), and Tyr189–propionate (red) distances.

In the second pose (Figure 3), anchoring of the substrate is performed instead through the interaction of the propionate group with Tyr189 (1.79 ± 0.32 Å) and the C2-hydroxyl group of the NADPH ribose (1.77 ± 0.20 Å). Compared to the first pose, the substrate lies 180° degrees flipped along the protochlophylide plane so that, although ring B still lies in the pocket defined by the inner surface of the flexible 225–250 domain and ring D lies above the NADPH nicotinamide ring, ring A in the second pose roughly occupies the position taken up by rings C and E in the first pose and vice versa. The keto group in the E ring is markedly less exposed to the solvent in this conformation than in the previous pose. In this pose, NADPH consistently remains farther from C_17_ (4.08 ± 0.48 Å) than in the previous conformation, and no proton donor is ever present close to C_18_, which strongly argues against a role for this conformation in the catalytic cycle. Quantum chemical computations on the reaction mechanism were therefore performed using the coordinates obtained from the simulation of the previous conformation. The snapshot taken at 100 ns was used for this purpose.

### DFT Exploration of the Reaction Mechanism Arising from Ground- or Excited-State Hydride Transfer from NADPH to PChlide

**Figure 4:**
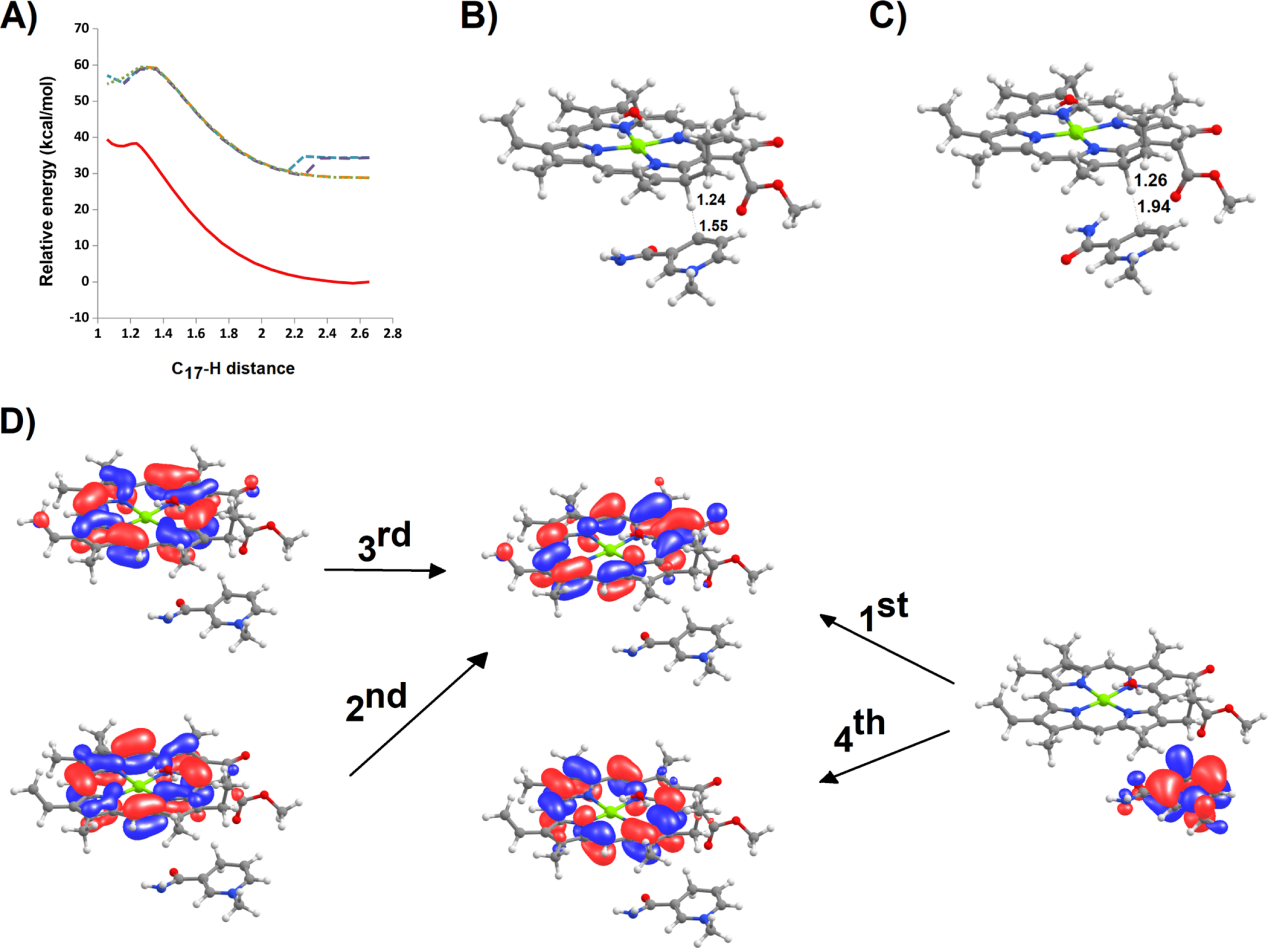
Hydride transfer from NADPH to PChlide. (A) Potential energy surfaces at the ground state (red) and the first four excited states (green, blue, violet, and orange, respectively). The excited energy surfaces depicted refer to the optimized structures of each excited state rather than to the energies of each excited state at the optimized ground state geometries. (B) Approximate structure of the transition state in the ground potential energy surface. (C) Approximate structure of the transition state in the first excited potential energy surface. (D) Orbitals involved in the first four excitations of the NADPH:PChlide system.

**Figure 5:**
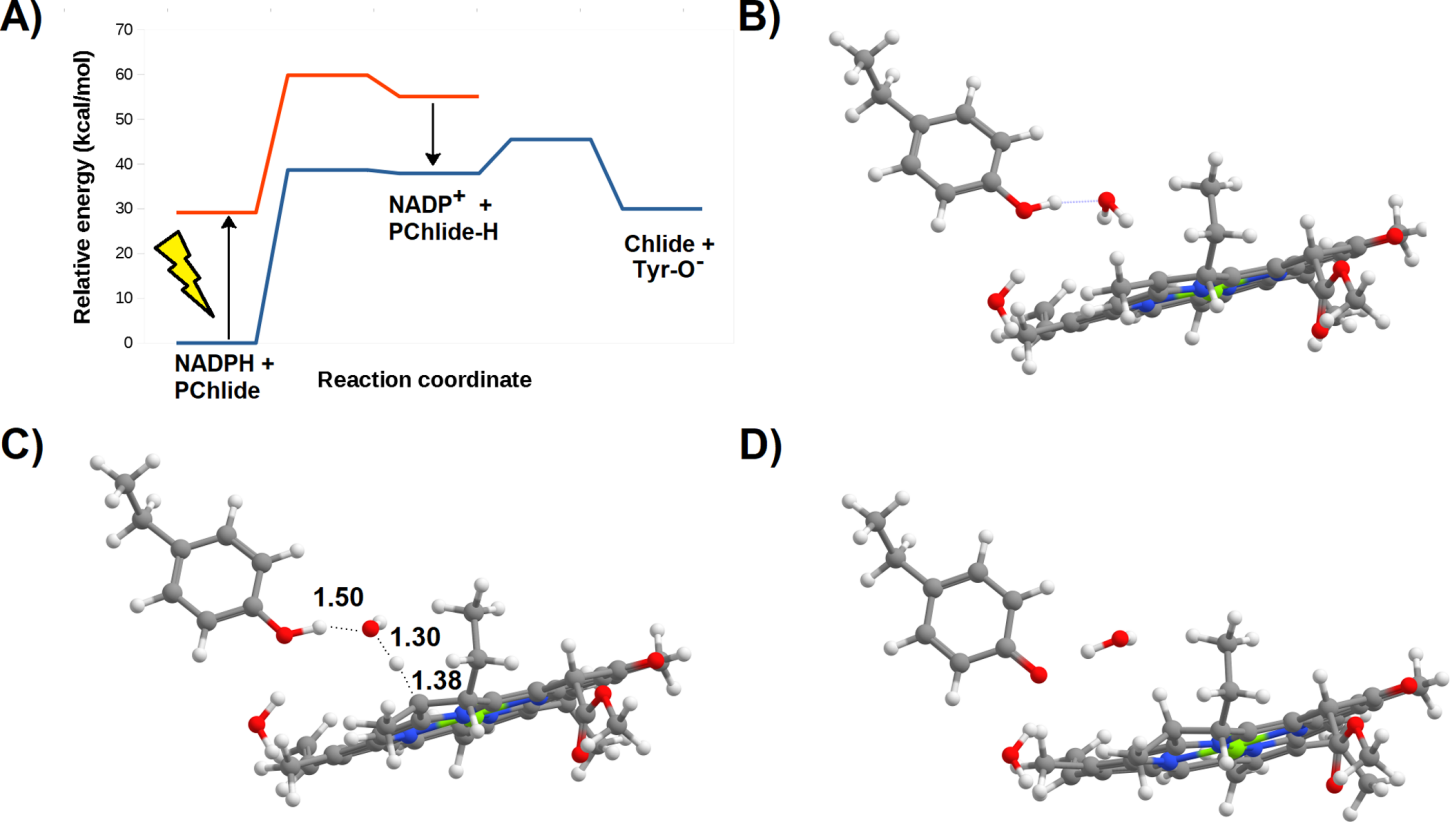
Potential energy surface in the pathway beginning with excited-state hydride transfer from NADPH to PChlide. (A) Relative reaction energies of the intervening intermediates and transition states. (B) Reactant state in the Tyr189:PChlide–hydride proton transfer step. (C) Transition state of the proton transfer step from Tyr189 to the hydride-reduced PChlide. (D) Final state of the proton-transfer state. All distances are in ångstrom.

In agreement with previous computational studies,^33,39^ our new DFT computations found that the activation energy of the ground-state hydride transfer from NADPH to protochlorophylide C_17_ is prohibitively high at 39.8 kcal mol^–1^ (Figure 4A,B). In the first four excited states (Figure 4A,D), this activation energy is lowered to 28.5–30.8 kcal mol^–1^, which is still well above the upper limit compatible with experimentally detectable reaction rates (for example, Eyring’s equation predicts that a reaction rate as low as 1 per hour requires an activation energy below 22.3 kcal mol^–1^) and considerably above the 8.8 kcal mol^–1^ determined experimentally.^33^ All excited-state potential energy surfaces are similar, and indeed, the energies of the second and third excited states become almost indistinguishable from those of the first and fourth excited states when the distance between the NADPH hydride and the receiving C_17_ atom in protochlorophyllide decreases below 2.1 Å. The geometries of the transition states in three out of four of these excited potential energy surfaces are quite similar to each other (Figure 4C), with the hydride much closer (1.25 Å) to the receiving C_17_ than to the donating nicotinamide C_4_ atom (1.95 Å), whereas in the remaining excited potential surface (the second one), the nicotinamide ring is significantly closer to PChlide, and the hydride is much closer to the midpoint of those two atoms (1.35 Å from C_17_ and 1.4 Å from the nicotinamide C_4_ atom). This difference is not, however, especially interesting, since in all four excited potential energy surfaces, the energies of these two structures are remarkably similar (<0.5 kcal mol^–1^), and therefore, the region around the transition state is quite flat. The return of the hydride to NADP^+^ is extremely fast, with activation energies below 2 kcal mol^–1^ in the ground state and below 5 kcal mol^–1^ in any of the four excited states. In this mechanism (Figure 5A), the transferred hydride will therefore promptly return to NADP^+^ unless the formed PChlide:H^–^ intermediate immediately acquires a proton, thereby yielding a stable Chlide. The DFT study of that step clearly shows, however, that proton transfer from Tyr189 to the PChlide:H^–^ (Figure 5B–D) is not as fast as required: the computed activation energy for the water-assisted transfer of that proton (7.6 kcal mol^–1^) yields a reaction rate 10^2^ to 10^4^ times slower than that of the return of the hydride to NADP^+^. Both the extremely high endergonicity of the hydride transfer and the difficulty in protonating the PChlide:H^–^ intermediate before the hydride returns to NADP^+^ very strongly suggest that the actual reaction mechanism should not proceed as a two-step process initiated by hydride transfer but rather as either a concerted process or a two-step process initiated by proton or single-electron transfer.

**Figure 6:**
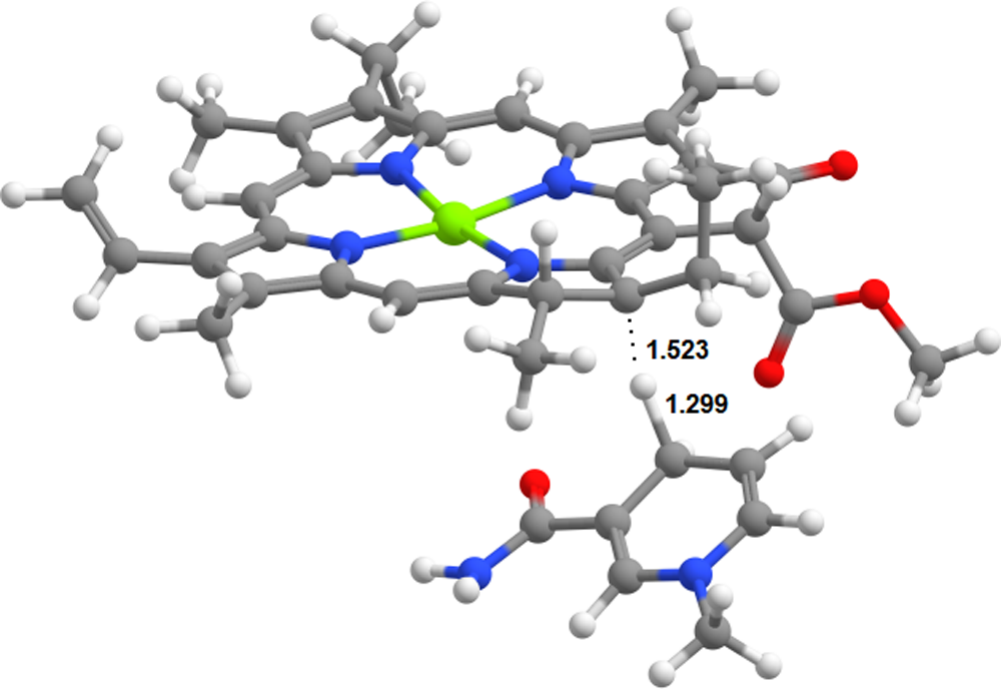
Transition state of the hydride transfer from NADPH to C_18_-protonated PChlide. Distances are in ångstrom.

**Figure 7:**
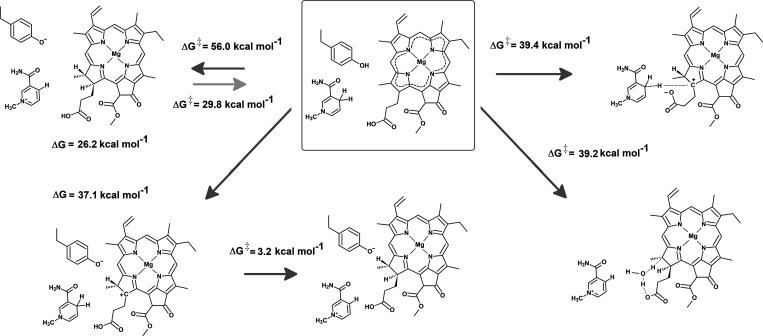
Analysis of the thermodynamic and kinetic feasibility of different ground-state mechanisms.

Such pathways are, however, not possible in the ground state. A two-step process initiated by proton transfer to C_18_ cannot occur because although subsequent hydride transfer from NADPH to a C_18_-protonated PChlide (Figure 6) proceeds extremely fast (with activation energies ranging between 1 kcal mol^–1^ (B3LYP/6-311G(+)(d,p)//B3LYP/6-31G(d)^19^) and 3.2 kcal mol^–1^(PBE0/6–311(partial+)G(d,p)//PBE0/6-31G(d); this work), the initial protonation of PChlide at the C_18_ position by Tyr189 is inaccessible in the ground state because it lies 45.3 kcal mol^–1^ above the neutral Tyr189:PChlide system. The addition of seven water molecules to the model system to facilitate charge delocalization can only reduce the endergonicity of this proton-transfer step to 37.1 kcal mol^–1^, which clearly establishes that this reaction is impossible in the ground state (Figure 7). On the other hand, simultaneous hydride transfer from NADPH to C_17_ and proton transfer from Tyr189 to C_18_ was also shown by our computations to be unfeasible in the ground state due to an extremely high activation energy of 56.0 kcal mol^–1^. The reaction is endergonic by 26.2 kcal mol^–1^. This value entails that the oxidation of the chlorophyllide product by NADP^+^ (with the concurrent return of the C_18_ proton to Tyr189) will have a high activation energy (56.0 – 26.2 = 29.8 kcal mol^–1^), which explains why the protein:Chlide:NADP^+^ formed in the end of the catalytic cycle does not decay back into reactants despite the thermodynamic spontaneity of the reverse reaction.

Alternative protonation of the critical C_17_=C_18_ bond by a putative protonated form of the propionic acid substituent present in the C_17_ position of PChlide also proved unfeasible despite the higher acidity of the carboxylic acid: earlier computations^19^ had already shown that in the absence of explicit water molecules, this proton transfer is slow and thermodynamically disfavored (activation energy of 24.7 kcal mol^–1^ and endergonicity of 19.8 kcal mol^–1^), and our new computations showed that the expansion of that computational model with additional water molecules created a water chain that further facilitated the return of the proton in C_18_ to the propionate. The C_18_-protonated intermediate was therefore no longer stable, and only concerted proton/hydride transfer mechanisms could occur. Two-dimensional scans of the potential energy surface detected two saddle points corresponding to two distinct partially concerted mechanisms. In one of the saddle points located 39.2 kcal mol^–1^ above the initial NADPH:Chlide-COOH state, hydride transfer is almost complete (the C_17_–hydride bond is 1.14 Å long), and the incoming proton (provided by a water molecule that has just abstracted a proton from the propionic acid) still lies 1.48 Å away from C_18_. In the other saddle point, which lies 39.4 kcal mol^–1^ above the reactants, the proton donated to C_18_ lies only 1.13 Å away, whereas the hydride lies 1.34 Å from C_17_ and 1.45 Å away from the nicotinamide. Not surprisingly, transferring a proton from the propionic acid to the PChlide:H^–^ (whether this molecule has been generated in the ground state or through an excitation) yields a lower barrier than that arising from proton transfer from Tyr189 to PChlide:H^–^ but is still unable to yield a mechanism compatible with the experimental observations due to the high activation energy of the hydride transfer from NADPH to PChlide. Finally, one-electron transfer from NADPH to PChlide is also impossible in the ground state due to the 48.4 kcal mol^–1^ difference in NADPH ionization energy and PChlide electron affinity.

### DFT Exploration of Novel Reaction Pathways

**Figure 8:**
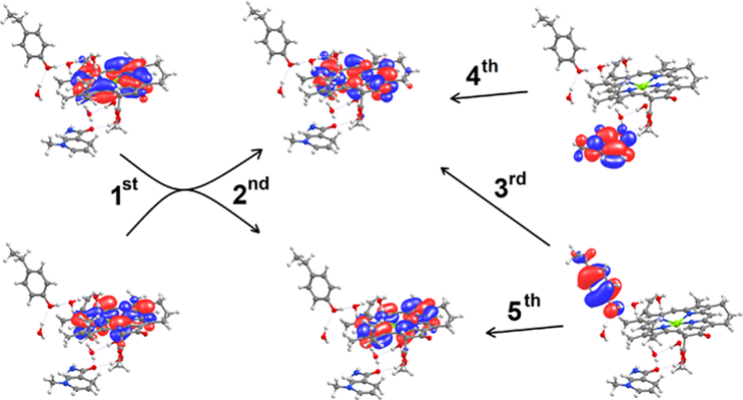
Orbitals involved in the five lowest-energy excitations in the NADPH/protochlorophyllide/Tyr189 system.

Our time-dependent DFT computations on the NADPH/substrate system described above showed that although PChlide-centered charge-separated states are indeed low-lying (as the second and third excited states), subsequent hydrogen transfer is prohibitively expensive since it proceeds with an activation energy of 28–30 kcal mol^–1^, which is far in excess of the experimentally computed barrier^33^ of 8.8 kcal mol^–1^. We therefore turned our attention to the analysis of a mechanism starting from an initial light-induced electron transfer from NADPH to the protochlorophyllide followed by proton transfer from the NADPH^+^ cation radical to the substrate anion radical, which has been proposed by Archipowa et al.^68^ to explain the transient absorption experimental data as arising from four individual electron (or proton) transfers. Two excited states characterized by complete charge transfer from NADPH to PChlide could indeed be found in our TD-DFT computations on the NADPH/PChlide system (Figure 4D). Exploration of these excited states’ potential energy surfaces on the larger NADPH/PChlide/Tyr189 system revealed that the transition state for the postulated transfer of the remaining H^+^:e^–^ from NADPH to the PChlide anion lies, like the transfers of the full hydride to the π–π* excited PChlide described earlier, more than 25 kcal mol^–1^ above the excited state-relaxed geometry of the NADPH^+^:PChlide^–^ ion pair, which is still far above the experimental activation energy. TD-DFT computations on this NADPH/PChlide/Tyr189 system revealed us the existence of two low-lying excited states characterized by a hitherto unsuspected excitation from an occupied π orbital in Tyr189 into an unoccupied π* orbital in the PChlide substrate (Figure 8).

**Figure 9:**
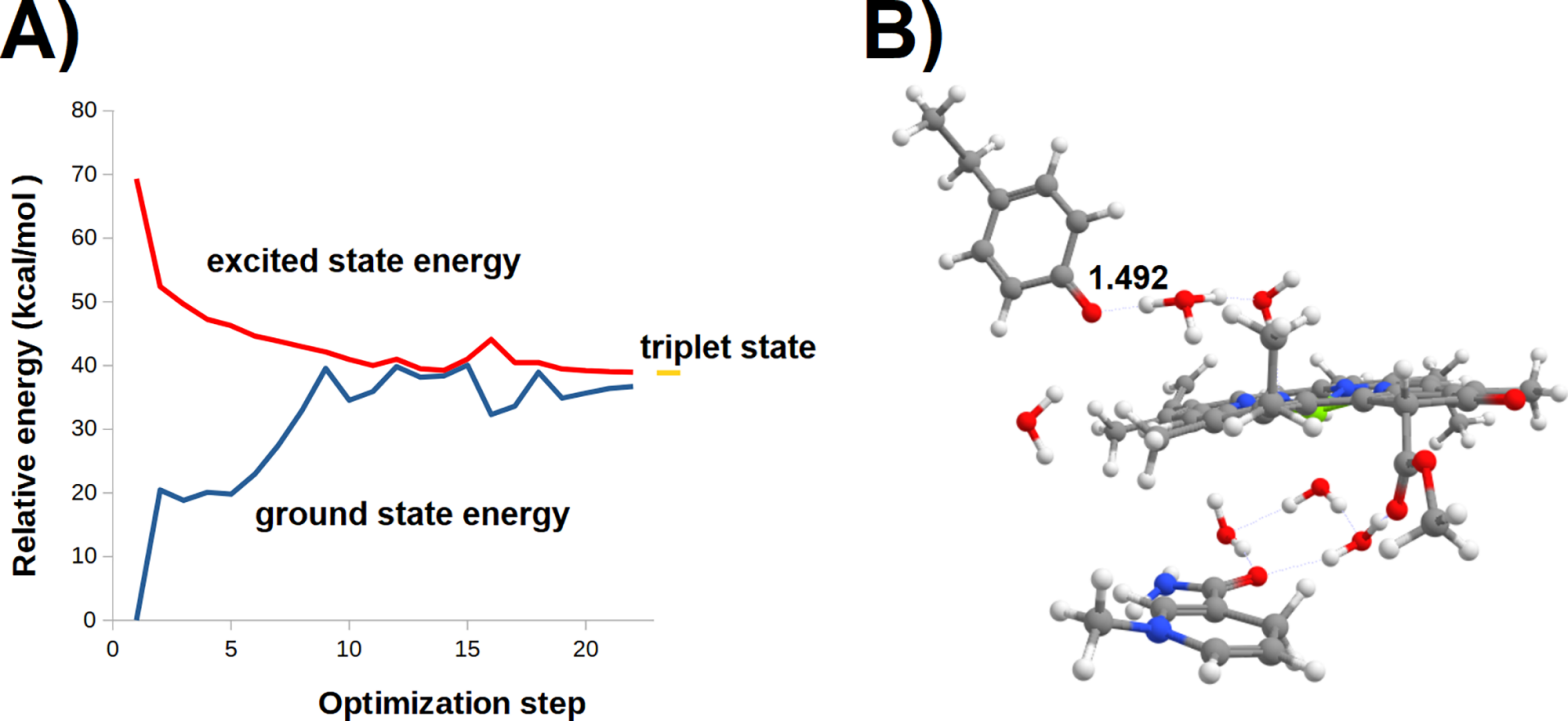
Evolution of the π Tyr189−π* PChlide excited state upon geometry optimization. (A) Excited- and ground-state energies of the system during geometry optimization. (B) Optimized geometry of the final Tyr189·:PChlide^–^:H_3_O^+^ system.

Optimization of this excited state showed spontaneous proton transfer from the one-electron-oxidized Tyr189 to the water molecule placed between this residue and the one-electron-reduced substrate (Figure 9B), yielding a neutral radical on the deprotonated Tyr189, a PChlide anion radical, and an H_3_O^+^ lying 2.15 Å from the substrate C_18_. The electronic structure of this excited state is effectively an open-shell singlet with an *S* = 1/2 electron in the substrate and an *S* = −1/2 electron on Tyr189, and its optimized structure corresponds to a point in the potential energy surface where the excited and ground states almost intersect. Should the system relax into the ground state, its geometry would then spontaneously return to the initial state and the reaction would not proceed. Three different factors concur to prevent this relaxation: first, the oscillator strength of the transition to the ground state (as computed by TDDFT) is extremely small, which entails a very low probability of fluorescence; additionally, this excited state has almost exactly the same energy as the corresponding triplet state produced by the inversion of the spin of the transferred electron (Figure 9A), which entails that (if spin-orbit coupling in the PChlide^–^ system is strong enough) the excited electron may spontaneously change spin after moving to PChlide, thereby rendering its return to Tyr189 impossible due to Pauli’s exclusion principle. Such a spin-flip of a putative internal charge-transfer state has indeed already been observed in both free protochlorophyllide and PChlide:LPOR:NADPH complexes upon excitation with 450 nm light.^69^ Finally, even in the absence of such a spin-flip, other amino acid side chains in the protein structure may be oxidized by the deprotonated Tyr189 radical, which would then increase the distance between the *S* = 1/2 and *S* = −1/2 electrons and prevent them from quickly recombining into the original electronic distribution.

**Figure 10:**
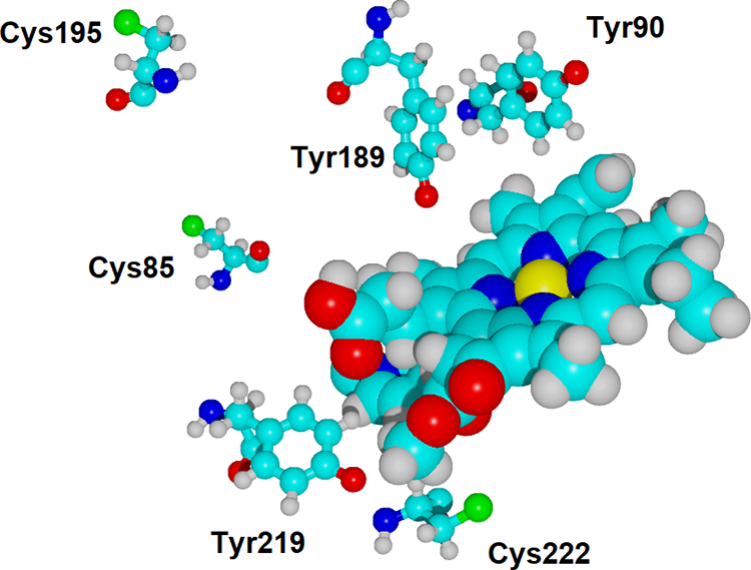
The geometric arrangement of substrate, Tyr189, and the five conserved Cys/Tyr residues present in its vicinity (as observed 100 ns throughout the MD simulation). Distances between side chains remain stable from *t* = 50 ns onward. Although the simulation was run with the physiological protonation states, side chains are depicted in their deprotonated forms, which are the ones susceptible to lose one electron to the deprotonated Tyr189 radical.

We have therefore computed the oxidation potentials of tryptophane, phenylalanine, cysteine (neutral and deprotonated), and tyrosine (neutral and deprotonated) side chains. Electron transfer from deprotonated Cys to a deprotonated Tyr189 radical was shown by these computations to be consistently favorable (from −21 to −19 kcal mol in the absence of explicit water molecules around Cys or Tyr, depending on the dielectric constant chosen for the medium, or −5.1 kcal mol when water molecules are explicitly included around Tyr to mimic its solvent-exposed status, whereas Cys is kept unsolvated to simulate its less-exposed state), whereas no other alternative was found to afford spontaneous electron transfers at any dielectric constants. Electron tunneling inside proteins is well known to be feasible when the electron donors and acceptors are closer than 16–18 Å,^70,71^ and inspection of the three-dimensional structures of the enzyme from *Synechocystis* (PDB:6R48) and *Thermosynechococcus elongatus* (PDB:6RNW) showed the presence of three conserved cysteine residues 10.5–13 Å from Tyr189 (Cys85, Cys185, and Cys 222) as well as two conserved tyrosine residues in close proximity to Tyr189 (Tyr90 at 5 Å and Tyr219 8 Å away) (Figure 10), which we therefore suggest may be involved in the ultimate stabilization of the electron-separated state initially generated through the excitation of one Tyr189 electron into the substrate. Computation of the activation energies of these electron transfers (Table 1) reveals a negligible barrier of 0.1 kcal mol^–1^ for the electron transfer from bare deprotonated cysteine to microsolvated radical deprotonated tyrosine, which (combined with the crystallographic Cys–Tyr distances) translates^70^ to an electron transfer rate of 10^7.1^ to 10^8.6^ s^–1^. The electron transfer from the deprotonated forms of Tyr90 and Tyr219 to the radical deprotonated Tyr189 also has a very small activation energy (2.3 kcal mol^–1^), entailing a very fast rate of 10^8.9^ to 10^9.5^ s^–1^.

**Table 1:** Activation Energies (in kcal mol^–1^) for the Electron Transfers between Unsolvated Deprotonated Cys and Different Microsolvated Tyrosine-Based Radicals^a^

		electron acceptor		
		Cys·	TyrOH·^+^	Tyr-O·
electron donors	Cys^–^	0.5	5.8	0.1
	Tyr-OH	45.8	4.2	41.9
	Tyr-O^–^	5.2	7.0	2.3

a All values were computed using a Marcus formalism at the PBE0/6-311(partial+)G(d,p)//PBE0/6-31G(d) level. Solvation effects were included (ε = 10).

Table 2 details average distances between these side chains and the estimated proportion (at pH = 7.0) of the respective deprotonated forms, which are thermodynamically capable of reducing deprotonated Tyr189 radicals. Application of the Moser–Dutton formula to these data shows that the “electron hole” in the deprotonated oxidized Tyr189 can very quickly transfer to either Tyr90 or Tyr219. Both Tyr90 and Tyr219 may quickly regain the electron they have released to Tyr189, but Tyr219 much more readily accepts it instead from Cys222. The spontaneity of this electron transfer can be strongly increased by the protein environment: continuum electrostatics computations of the complete protein show that, even if the intrinsic redox potentials of deprotonated Tyr and deprotonated Cys were initially assumed to be the same (instead of favoring the deprotonated Cys radical by 5.1 kcal mol^–1^ as in our quantum computations), the computed redox potential of the deprotonated Cys222 would be lowered by the protein environment by between 95 mV (at pH 8) and 110 mV (at pH 6) relative to that of the deprotonated Tyr189, which further confirms the high spontaneity of our proposed electron transfer from Cys222 to the deprotonated Tyr189 radical.

**Table 2:** Estimation of the “Electron Hole” Transfer Velocities (*v*_ET_) between Tyr189 and Surrounding Tyr/Cys Residues^a^

electron donor	electron acceptor	distance (Å)	log *k*_ET_	relative abundance of the deprotonated form of the electron donor	log *v*_ET_
Tyr90	Tyr189-O·	3.9 ± 0.4	11.4	3.9 × 10^–3^	**9.0**
Tyr219	Tyr189-O·	9.0 ± 0.6	8.4	4.4 × 10^–3^	**6.0**
Cys85	Tyr189-O·	14.2 ± 0.6	6.4	1.5 × 10^–4^	2.6
Cys195	Tyr189-O·	11.9 ± 0.8	7.8	3.0 × 10^–5^	3.3
Cys222	Tyr189-O·	13.4 ± 1.0	6.9	1.6 × 10^–4^	3.1
Tyr189	Tyr90-O·	3.9 ± 0.4	11.4	2.3 × 10^–2^	**9.8**
Cys222	Tyr90-O·	16.2 ± 1.1	5.2	1.6 × 10^–4^	1.4
Tyr189	Tyr219-O·	9.0 ± 0.6	8.4	2.3 × 10^–2^	6.7
Cys222	Tyr219-O·	4.9 ± 0.6	12.0	1.6 × 10^–4^	**8.2**

a *v*_ET_ values were computed by multiplying electron-transfer rates (k_ET_, obtained through the Moser–Dutton equation) by the amount of the reactive deprotonated form of the electron-donating amino acid side chain (computed through a Monte Carlo procedure based on the results from continuum electrostatic calculations). All distances shown are the average values measured between *t* = 100 ns and *t* = 300 ns of our simulation. The geometric arrangement of these residues relative to the substrate is shown in Figure 10. The highest transfer velocities are highlighted in bold.

**Figure 11:**
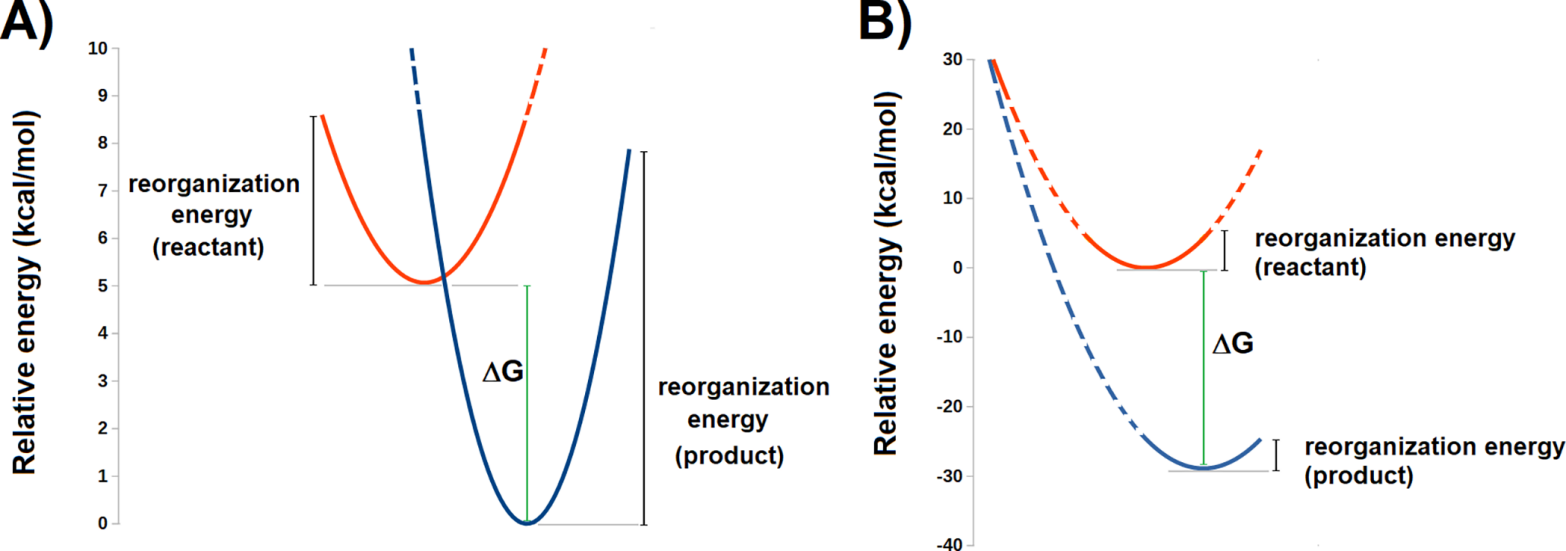
Marcus parabolas for the calculation of the activation energies of electron-transfers from (A) Cys^–^ to the deprotonated neutral Tyr radical and (B) PChlide^–^ to deprotonated Cys· neutral radical. The activation energy can be determined by comparing the initial state with the point where both parabolas intersect. A Marcus inverted region, where increased exergonicity leads to increased activation energies, is apparent in panel B due to the large value of the exergonicity relative to the respective reorganization energies.

The migration of the electron hole to Cys222 prevents the reaction from running backward, since the extra electron now present in the PChlide can no longer return to Tyr189, which is occupied by the electron previously released by Tyr219/Cys222. The premature unproductive termination of the reaction through transfer of the extra electron from PChlide^–^ to the oxidized Cys222· would be prevented both by the spin-flip described above (which would have aligned the spins in PChlide^–^ and Cys222·) and by the presence of a Marcus inverted region caused by the high exergonicity of the putative electron transfer between both species (Figure 11 and Table 3). Evaluation of the solvent contribution to these reorganization energies through molecular dynamics simulations of the corresponding PChOR:NADPH:substrate systems using Zhou and Szabo’s method^62^ confirms the favorability of the electron transfer from Cys222^–^ to the deprotonated Tyr189 radical, whose activation energy only increases by 1.9 kcal mol^–1^ when including the solvent reorganization.

**Table 3:** Activation Energies (in kcal mol^–1^) for the Electron Transfers from One-Electron-Reduced (Proto)chlorophyllide Species to Cys- or Tyr-Based Radicals^a^

	electron acceptor		
electron donor	Cys-S·	Tyr-OH·^+^	Tyr-O·
one-electron-reduced PChlide	35.7	19.0	5.5
C18-protonated one-electron-reduced PChlide	0.3	4.8	<0.1
C20-protonated one-electron-reduced PChlide	<0.1	4.6	<0.1
one-electron-reduced Chlide	5.6	12.6	2.6

a All values were computed using a Marcus formalism using PBE0/6-311(partial+)G(d,p)//PBE0/6-31G(d). Solvation effects were included (ε = 10).

This proposal is consistent with previous mutagenesis studies, which showed that the replacement of Cys222 by Ser^72^ of the A_696_ intermediate, decreased the reaction rate up to 15-fold, and greatly decreased the maximum amount of product generated. The observation of residual Chlide production in the mutant, where the characteristic 696 nm absorption of A_696_ intermediate (known to be produced after subsequent hydride transfer) is not detected, could then be explained by positing that this A_696_ species does not simply correspond (as usually assumed) to the hydride-reduced PChlide but is instead due to the interaction of the one-electron-reduced PChlide with the radical form of the deprotonated Cys222·, whereas in the C222S mutant, the radical electron hole would remain in Tyr219 and not give rise to that absorption.

We performed the study of the subsequent reactions in a truncated system missing the Tyr189 side chain, both because of the extremely high likelihood of the transfer of the radical to Cys222 and because the computational exploration of potential energy surfaces in the excited state is extremely demanding both in time and computational resources as well as fraught with difficulties. The electronic structure of this truncated *S* = 1/2 system is much more amenable to SCF convergence than that of the open-shell excited state singlet, and the following steps can then be simulated as ground-state proton transfer from the H_3_O^+^ generated in the previous step to the PChlide anion radical, followed by a hydride transfer to the C_18_-protonated PChlide anion radical.

**Figure 12:**
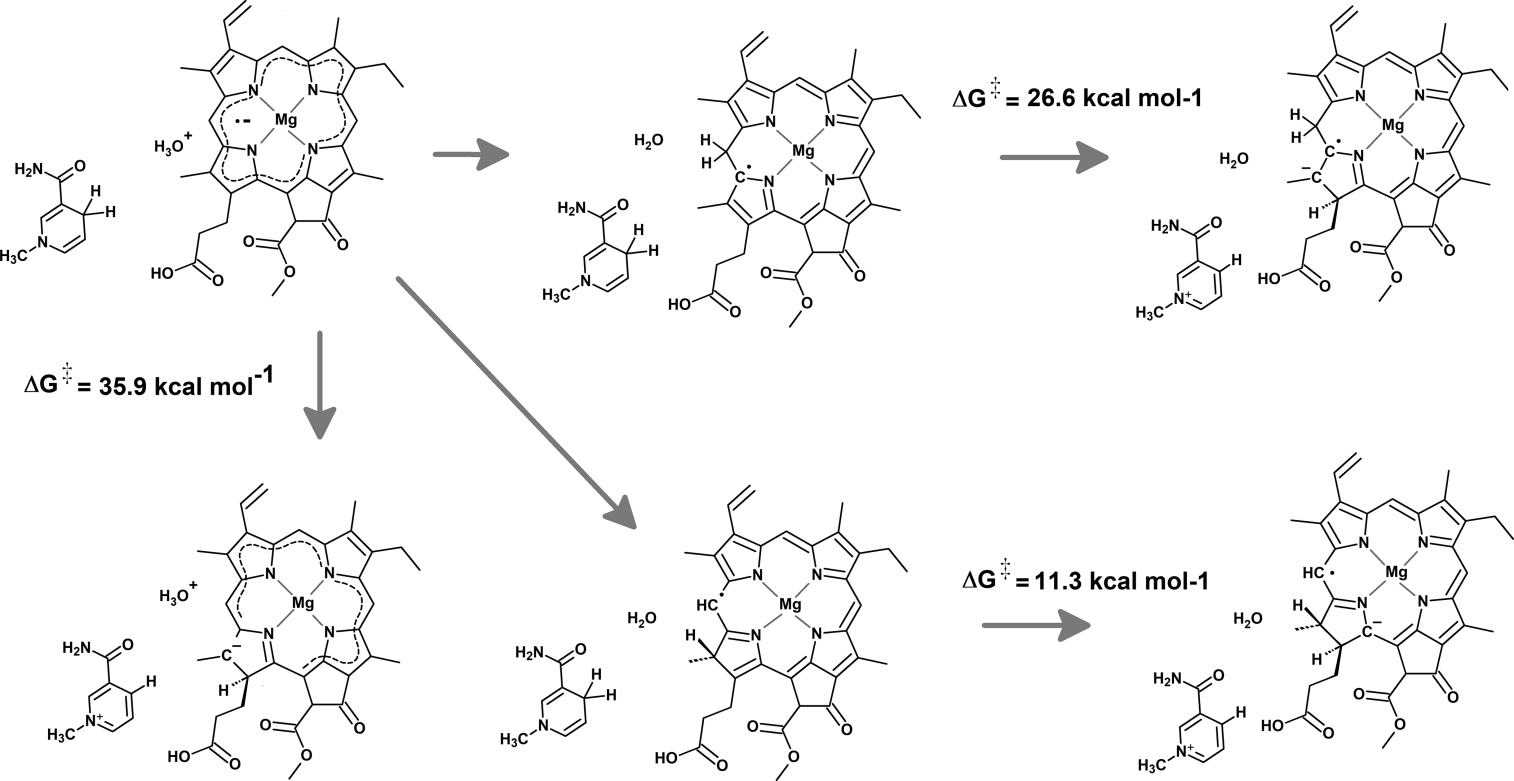
Possible reaction pathways of one-electron-reduced PChlide toward H_3_O^+^ and NADPH. The two protonation steps shown occur without a barrier.

The decrease in system size provided by the elimination of the Tyr189 residue afforded the opportunity to include a few extra water molecules to improve the description of the solvation of H_3_O^+^ while preserving computational tractability. In this improved system, we found that the separated H_3_O^+^ and the one-electron-reduced substrate cannot coexist; instead, the substrate will become protonated either on its C_18_ or C_20_ atoms. The two states are connected by a transition state containing a Zundel ion (H_3_O^+^:H_2_O) hovering above the C_18_ atom, 33.4 kcal mol^–1^ above the C_18_-protonated product and 36.5 kcal·mol^–1^ above the C_20_-protonated alternative. These computations show that the proton may therefore spontaneously transfer to either C_18_ or C_20_ and is then kinetically trapped in that position. Although this truncated model is insufficient to explain why the C_18_ product is always observed to be formed instead of the C_20_-protonated alternative, we have noticed that the computation of the light-induced deprotonation of Tyr189 is sensitive to the precise initial orientation of the Tyr189 and the positions of added waters, which can in some models yield an H_3_O^+^ hovering above C_18_ and, in others, an H_3_O^+^ much closer to the C_20_ atom. It is therefore likely that the precise reaction outcome is governed by dynamic factors that stabilize a Tyr189 orientation conducive to the formation of the C_18_ product. An alternative mechanism involving direct hydride transfer from NADPH to the substrate anion radical before the proton transfer is impossible as it is prevented by an activation energy of 35.9 kcal mol^–1^. The immediate product obtained after light-induced electron transfer from Tyr189 to the substrate is therefore shown by these computations to be either a C_18_-protonated or a C_20_-protonated neutral PChlide radical (Figure 12). Interestingly, analysis of the reorganization energies in Tables 3 and 4 shows that premature radical quenching through electron transfer from the protonated C_18_-protonated (or C_20_-protonated) one-electron-reduced PChlide to the deprotonated Cys222 radical may be thermodynamically and kinetically possible, provided that the unpaired electrons in Cys222/protonated PChlide have opposite spins. Interestingly, the application of Zhou and Szabo’s method^62^ to this electron transfer to evaluate the solvent contribution to the reorganization energy of this electron transfer showed that this quenching route becomes disfavored: its moderate inner-shell activation energy is dwarfed by a massive destabilization of 17.0 kcal mol^–1^ due to the outer-shell reorganization. Productive continuation of the reaction sequence therefore does not necessarily require that the extra electron in the PChlide ring has undergone a spin inversion.

**Table 4:** Activation Energies (in kcal mol^–1^) for the Electron Transfers from Cys or Tyr Side Chains to Neutral or Cationic (Proto)chlorophyllide Species^a^

		electron acceptor			
		PChlide	C18-protonated PChlide	C20-protonated PChlide	Chlide
electron donors	Cys-S^–^	64.6	2.1	4.0	35.7
	Tyr-OH	88.0	46.6	48.6	82.7
	Tyr-O^–^	39.5	6.9	9.1	37.7

a All values were computed using a Marcus formalism using PBE0/6–311(partial+)G(d,p)//PBE0/6-31G(d). Solvation effects were included (ε = 10).

Hydride transfer from NADPH to the putative C_20_-protonated neutral radical is endergonic by 16.9 kcal mol^–1^ and has a high activation energy of 26.6 kcal mol^–1^. The C_20_-protonated neutral radical intermediate therefore seems to be a kinetic dead end and, should the previous reaction step generate a C_20_-protonated product, the reaction will not proceed further; at most, the proton will eventually return to Tyr189 when the extra electron present in the ring is recuperated by the Cys222 radical, yielding a futile cycle.

In contrast, hydride transfer from the NADPH to the C_18_-protonated neutral radical is computed to be exergonic by 11.0 kcal mol^–1^ and to proceed very easily with a barrier of only 11.3 kcal mol^–1^, which agrees quite well with the 8.8 kcal mol^–1^ barrier determined experimentally^33^ for the formation of the A_696_ intermediate. Neither the differences in stability between the products or the activation energies of the reactions arising from hydride transfer to the C_18_-protonated and C_20_-protonated species are surprising because in the latter instance, the replacement of the C_16_–C_17_ = C_18_–C_19_ = C_20_–C_1_ conjugated segment by a nonconjugated (C_16_–C_17_–C_18_ = C_19_–C_20_–C_1_) single bond breaks the extended conjugated π-system, whereas converting only the C_17_–C_18_ double bond into a single bond has a much smaller effect on the length of the conjugated system. The product of this step is a one-electron-reduced chlorophyllide, and the termination of the reaction will then proceed through the return of the extra electron to the deprotonated Cys222· radical. Tables 3 and 4 show that this quenching reaction has a very favorable activation energy (5.6 kcal mol^–1^) and is irreversible (due to the 35.7 kcal mol^–1^ barrier of the opposite reaction), and the short distance between Cys222 and the substrate (7.3 ± 0.6 Å) further facilitates its progress at very high rates provided that a new spin-flip has occurred so that both electrons have opposite spins and that the reaction is therefore no longer spin-forbidden. The additional solvent contribution to this activation energy, as evaluated through Zhou and Szabo’s method,^62^ is indistinguishable from zero.

Experimental analysis of the “dark” steps following the hydride transfer has shown that at least two individual steps^73^ are involved before the formation of the characteristic 671 nm absorption band attributed to the LPOR-NADP^+^-Chlide ternary product complex, one of which is subject to a solvent isotope effect when the reaction is performed in deuterated water^33^ and has been previously attributed to proton-transfer to hydride-reduced PChlide. In our model, the proton transfer takes place before the hydride transfer, and the observed solvent isotope effect must therefore be attributed to a different event. We propose that this dependence is instead due to the uptake of a solvent proton by the deprotonated Cys222 after this side chain has received the extra electron from Chlide^–^ (and accompanying conformational change due to the change in charge distribution and flexibility of that region of the protein) to regenerate the active site for the next round of catalysis. In this interpretation, the intermediate (absorbing at 681 nm and fluorescing at 684 nm^73^) observed between the A_696_ intermediate and the A_671_ product would be a LPOR-NADP^+^-Chlide ternary complex bearing an anionic Cys222, and the A_671_ product would be the LPOR-NADP^+^-Chlide ternary complex bearing a neutral Cys222. This is consistent both with the lack of a solvent isotope effect in the Cys222Ser mutant^72^ and with the observation of a significant influence of solvent viscosity for these steps (i.e., conformational changes), which (due to the close proximity of Tyr189 to solvent and substrate throughout our molecular dynamics model of the ternary complex) would be hard to explain in a model attributing this step to proton transfer from Tyr189 to substrate.

## Discussion

**Figure 13:**
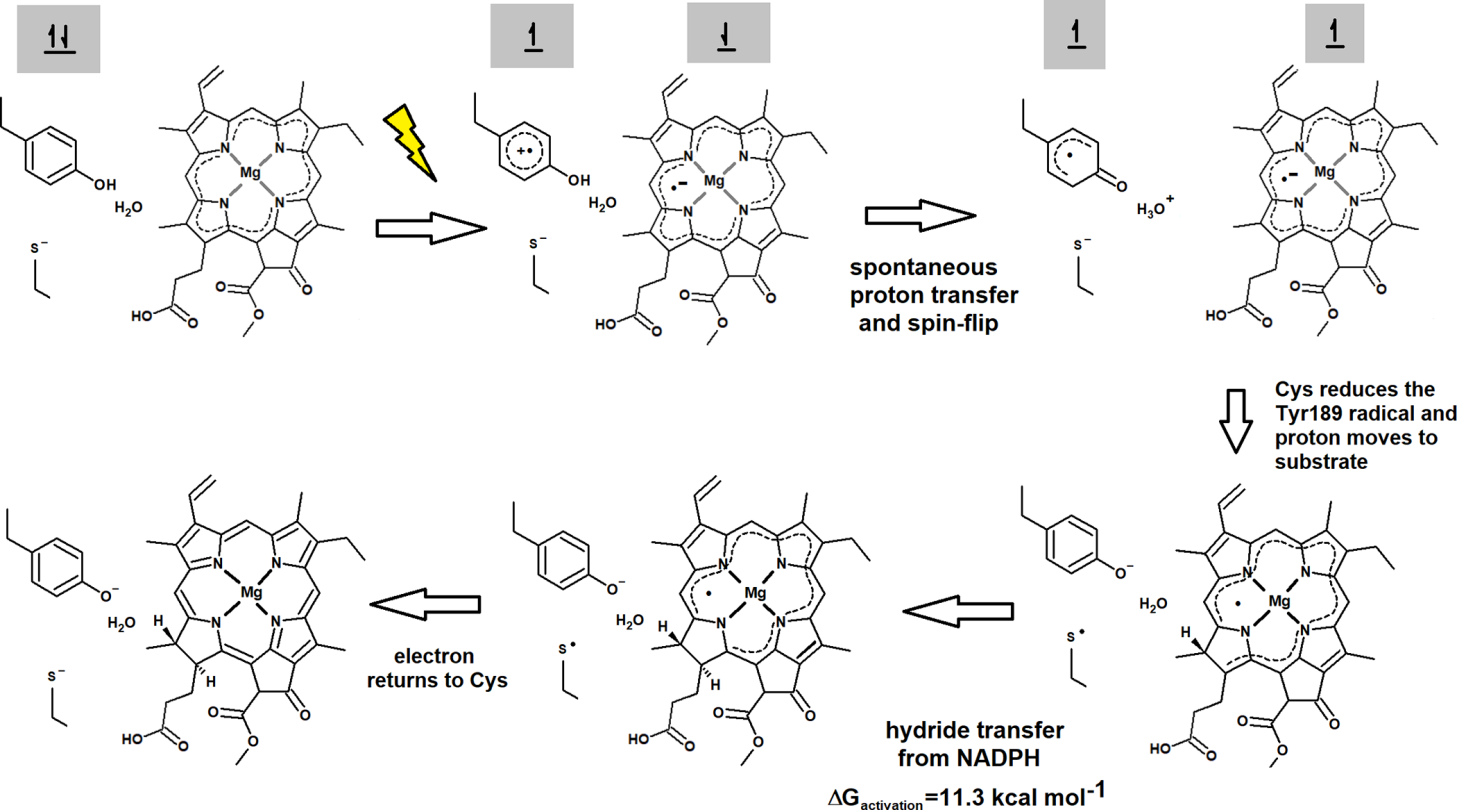
Proposed reaction mechanism. Spin orientation in the initial reaction stages is depicted in the gray boxes.

The pathway (Figure 13) described above is very different from previous proposals,^74^ which generally start with either an initial hydride transfer from NADPH to an excited substrate^68,69^ or a single electron transfer from NADPH to substrate.^72^ It is not, however, devoid of experimental support since early electron-spin resonance studies of this reaction^75,76^ mechanism reported the observation of radical signals identical to the ones generated upon chemical reduction of protochlorophyllide to its anion radical state. Similar signals have, in other studies,^29^ been attributed to PChlide-based radical cations. The peak-to-peak linewidth of radical signals has been claimed to be sufficiently different to tell radical cation EPR signals from radical anions in chlorophyll,^77^ but small linewidths (usually attributed to the radical cations) have also been found in Mg-porphyrins in their radical anion state.^78^ The precise attribution of the radical signals observed in the light-dependent protochlorophyllide reductase, their possible roles in the mechanism, and whether they are indeed, as suggested by our computations, due to one-electron reduced PChlide, one-electron-reduced Chlide, or one-electron-reduced C_18_-protonated PChlide must therefore await further experimental examination. The postulated one-electron-reduced forms of PChlide present in our mechanism also suggest the possibility of further side reactions with possible biological consequences since the excess of electronic charge in their extended conjugated systems increases their susceptibility to oxidation.^79^ According to our computations of activation energies for the hydride transfer and electron-transfer steps, the one-electron C_18_-protonated PChlide has the longest half-life (due to its activation energy of 11.3 kcal mol^–1^), whereas the one-electron reduced PChlide reacts with H_3_O^+^ without a barrier and the one-electron-reduced Chlide formed after hydride transfer must face a 5.6 kcal mol^–1^ barrier to return its extra electron to Cys222. We have therefore computed the activation energies of the electron-transfer reactions from these species to O_2_ to ascertain the likelihood of any of them generating superoxide, thereby terminating the reaction mechanism (Table 5). Electron transfer from the critical C_18_-protonated PChlide intermediate is thermodynamically and kinetically disfavored, whereas both Chlide^–^ and PChlide^–^ are prone to donate an electron to O_2_. In spite of the very small activation energies of these latter processes, we expect net superoxide production and premature reaction termination to be residual due to the barrier-less reaction of PChlide^–^ toward H_3_O^+^, which leads to its unavailability to react with oxygen and to the almost barrier-less activation energy for a subsequent electron-transfer from any superoxide (generated from Chlide^–^) to the Cys222 radical.

**Table 5:** Activation Energies (in kcal mol^–1^) for the Electron Transfers between Oxygen and One-Electron-Reduced Species^a^

electron donor	electron acceptor	activation energy (kcal mol^–1^)
PChlide^–^	O_2_	0.4
C_18_-protonated PChlide^–^	O_2_	9.2
Chlide-	O_2_	0.2
O_2_^–^	PChlide	18.7
O_2_^–^	C_18_-protonated PChlide	0.4
O_2_^–^	Chlide	19.7
O_2_^–^	Cys-S·	<0.1

a All values were computed using a Marcus formalism using PBE0/6–311(partial+)G(d,p)//PBE0/6-31G(d). Solvation effects were included (ε = 10).

**Figure 14:**
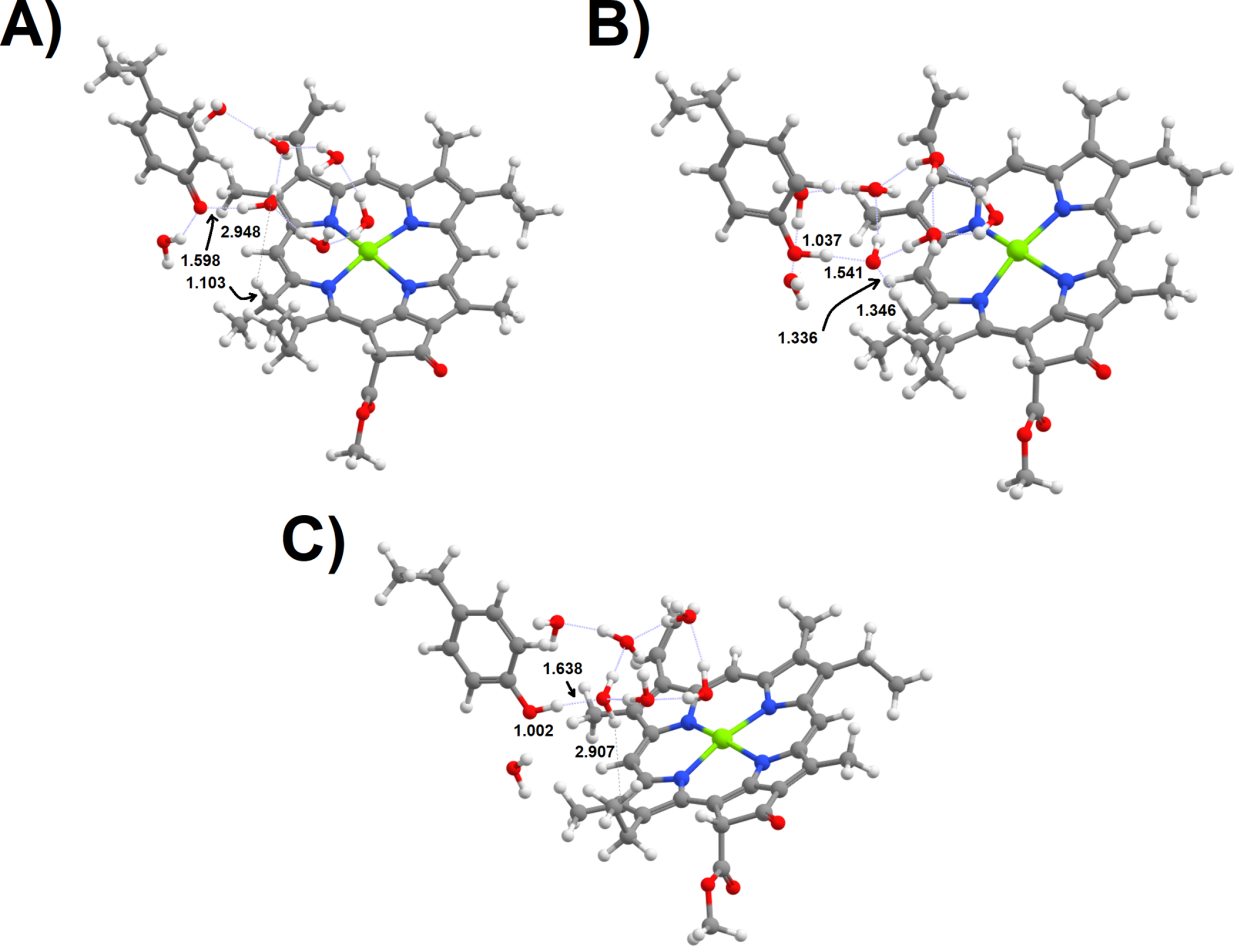
The unproductive reaction that is prevented by proton transfer from Lys193 to Tyr189: (A) reactant (deprotonated Tyr189 + C_18_-protonated, one-electron reduced PChlide), (B) transition state, and (C) product (neutral Tyr189 + one-electron reduced PChlide).

The computations described above do not yet explain why the active site residue Lys193 is essential for the reaction mechanism.^80−82^ We have therefore analyzed how the postulated protonation of Tyr189 by Lys193^80,81^ might help overcome an eventual unproductive side reaction. This proton transfer was revealed by additional DFT computations to proceed with a negligible barrier (<2 kcal mol^–1^), which entails that upon light-induced electron and proton transfer from Tyr189 to the substrate and subsequent electron transfer from Cys222 to Tyr189, the tyrosine can be readily returned to its initial neutral state. This is important because if Tyr189 had remained deprotonated, it might have been able to abstract the extra proton from the C_18_-protonated one-electron substrate, thus undoing the previous reactions steps. Indeed, computations on that system showed that this counterproductive reaction (Figure 14) is exergonic by 20.4 kcal mol^–1^ and has an activation energy of only 9.0 kcal mol^–1^. Despite its small magnitude, this barrier is much larger than that computed for the protonation of Tyr189 by Lys193. In the wild-type enzyme, therefore, Lys193 prevents this unproductive pathway from taking place and enables the C_18_-protonated one-electron-reduced PChlide to survive until it receives the hydride from NADPH.

## Conclusions

In a previous work^39^ (using smaller models and lacking excited-state geometry optimizations), we reported high barriers to the hydride-transfer step from NADPH to PChlide, although it went unnoticed at the time that those barriers were incompatible with the experimentally measured barrier of 8.8 kcal mol^–1^. The present work clearly establishes that such a hydride transfer is not feasible either in the ground state or in the excited state and also ruled out the possibility of a sequential electron/hydrogen/proton transfer initiated through light-induced one-electron oxidation of NADPH by PChlide. Alternatively, the computations described herein suggest a light-induced electron transfer from Tyr189 to PChlide, which strongly increases the acidity of Tyr189 and enables the transfer of a proton to the C_18_ (or C_20_) position of PChlide. The electron hole generated in Tyr189 quickly transfers to the strictly conserved Cys222 through the (also completely conserved) Tyr219 residue, after which the “back-reaction” can no longer take place. Hydride transfer from NADPH to the C_17_ position then takes place (with a computed barrier quite close to the experimental one), followed by the return of the extra electron in the substrate to the deprotonated Cys222 radical and the reprotonation of this residue by bulk solvent. A spin-flip of the extra electron transferred from Tyr189 to PChlide prevents unproductive futile cycling from prematurely terminating the reaction. Beyond its excellent agreement with the experimentally measured activation energy, this new model also appears to be consistent with a wide variety of experimental observations that have heretofore been interpreted in a different way and suggests new experimental tests for the detection of the new postulated intermediates in this intriguing enzyme.

## Supporting Information Available

Coordinates of all species described in this study (ZIP)

## Notes

Complete inputs and outputs for the quantum mechanical computations have been deposited at dx.doi.org/10.6084/m9.figshare.13636670
